# Investigating Ultrasonic Pulse Velocity Method for Evaluating High-Temperature Properties of Non-Sintered Hwangto-Mixed Concrete as a Cement Replacement Material

**DOI:** 10.3390/ma16031099

**Published:** 2023-01-27

**Authors:** Wonchang Kim, Hyeonggil Choi, Taegyu Lee

**Affiliations:** 1 Department of Fire and Disaster Prevention, Semyung University, Choongbuk 27136, Republic of Korea; 2 School of Architecture and Civil Engineering, Kyungpook National University, Daegu 41566, Republic of Korea

**Keywords:** non-sintered hwangto, ultrasonic pulse velocity, compressive strength, prediction model of compressive strength, high temperature

## Abstract

Research on alternative cement materials is active worldwide, and in terms of fire safety, research on the evaluation of high-temperature properties of alternative materials is very important. Studies on concrete mixed with hwangto have been conducted by several researchers, but studies on high-temperature properties are lacking. Therefore, in this study, we evaluated the mechanical properties of concrete by partially replacing cement with non-sintered hwangto (NSH) at high temperatures. Normal concrete without NSH mixing and non-sintered hwangto concrete (NSHC) with HNT replacement were prepared as the specimens. The W/B of the concrete was set to 41 and 33, whereas the NSH replacement ratio was 15 and 30% of the cement. The target heating temperatures were set to 20, 100, 200, 300, 500, and 700 °C, and the heating rate was maintained at 1 °C/min. The following were calculated to evaluate the mechanical properties of the specimens: mass loss, compressive strength, ultrasonic pulse velocity (UPV), and modulus of elasticity. After analyzing the correlation between residual compressive strength and UPV, we proposed a compressive strength prediction model using different values of W/B for NSHC. Experimental results suggest that mass loss (%) shows a decreasing trend as NSH increases. In terms of residual compressive strength, residual compressive strength at W/B 41 increased with NSH replacement, whereas residual compressive strength values for W/B 33 were observed regardless of NSH replacement. Residual UPV showed a similar trend, regardless of the NSH replacement ratio, and residual modulus of elasticity was low at all W/B ratios as NSH replacement increased. A linear equation with a high correlation coefficient (R^2^) was proposed to predict compressive strength, and the linear value of W/B 41 was slightly higher than that of W/B 33.

## 1. Introduction

In line with the global efforts toward reducing CO_2_ emissions, methods that reduce the use of cement and cement replacement materials have attracted significant attention [1,2]. Regarding cement replacement materials, previous studies have investigated natural minerals, such as diatomite and clay, as well as byproducts from processing in industrial sectors such as the steelmaking industry (such as fly ash, blast furnace slag, and red mud) [3,4,5,6,7]. Hwangto, as a cement replacement material, accounts for approximately 10% of the Earth’s surface, and it is a readily available material [8]. The chemical composition of hwangto includes SiO_2_ and Al_2_O_3_, making it similar to admixtures used in cement. Therefore, hwangto has been used as cement replacement material for CO_2_ reduction, particularly in Asian countries such as China and the Republic of Korea [9,10,11,12,13,14].

However, the existing research on hwangto as a building material primarily focuses on strength development mechanisms and construction at room temperature, and some previously reported studies have discussed the advantages of high-temperature properties of hwangto itself. [15,16]. In previous studies, thermogravimetric (TG) analyses (TG and differential thermal analysis) of hwangto revealed a total of three peaks, with two of these peaks caused by the evaporation of free and absorbed water appearing at temperatures below ~140 °C [17,18]. A peak representing kaolinite dehydrogenation in the 400–600 °C temperature range and another peak associated with calcite and illite decarbonization at temperatures above 600 °C were also reported [19]. At temperatures above 200 °C, the connection between the voids is improved, owing to the firing and shrinkage of hwangto minerals [20], and at 900 °C, most mineral particles (such as iron and aluminum minerals) undergo melting and adsorption during the cooling process after high-temperature exposure, increasing the contact between the mineral particles [21]. Furthermore, some minerals (such as Al_2_O_3_, CaO, and MgO) found in hwangto exhibit thermal expansion in the temperature range of 20–500 °C, which decreases the interlayer spacing, thereby reducing porosity [22], and at temperatures above 500 °C, the density increases with increasing contact between hwangto particles [23].

**Figure 1:**
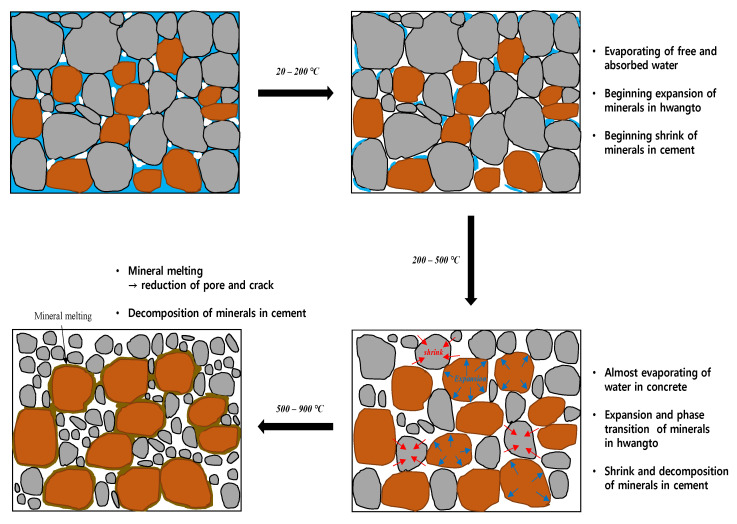
Mechanism of hwangto in concrete at high temperatures.

When a natural mineral such as ‘hwangto’ is mixed, it shows a lower strength development compared to 100% cement mixed concrete. In particular, the strength and durability of concrete that has been subjected a high temperature in the event of a fire are expected to decrease further. Therefore, engineers need to accurately and quantitatively evaluate concrete mixed with hwangto after a fire. However, as mentioned above, hwangto itself has an advantage under high-temperature conditions, and it is believed that mixing hwangto with concrete will have a positive effect. Figure 1 schematically shows the expected mechanism of hwangto when combined with cement based on the high-temperature properties of the existing hwangto. In addition, in order to evaluate these mechanisms, the ultrasonic pulse velocity (UPV) method was used in this study.

**Figure 2:**
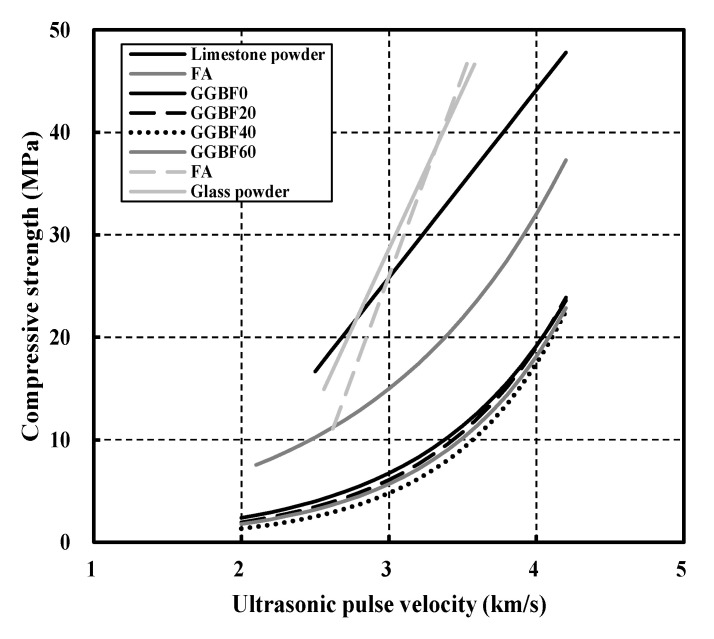
Previously proposed equations for estimating compressive strength by ultrasonic pulse velocity [26,27,28,29].

Researchers previously conducted a study using UPV to evaluate the internal condition and degradation of concrete (Figure 2 and Table 1). In general, researchers propose a strength prediction model by analyzing the correlation between the compressive strength and the UPV in concrete. However, because different compressive strengths and UPVs occur depending on the type of material mixed with concrete and the substitution rate of the mixture, additional strength prediction equations need to be proposed through experiments to accurately predict the strength of NSHC [24,25].

In this study, we investigated the high-temperature properties of concrete using hwangto as a cement replacement material, and we proposed a prediction model for compressive strength to enable the evaluation of degradation in concrete properties using UPV analysis [26,27,28,29]. Additionally, to analyze the effect of cement content and unit non-sintered hwangto (NSH) content on UPV, the water/binder (W/B) ratio and NSH replacement were analyzed at various ratios.

## 2. Materials and Methods

### 2.1. Experimental Outline

Table 2 presents the experimental parameters of this study. Normal concrete (NC) without non-sintered hwangto and non-sintered hwangto concrete (NSHC) mixed with non-sintered hwangto were chosen as the experimental specimens.

The W/B ratio was set to 0.33 and 0.41, and residual mechanical properties were evaluated with NSH mixed at different W/B ratios after high-temperature exposure. Cylindrical specimens (*ϕ* 100 × 200 mm) were prepared, and the specimens underwent water curing for 28 days, followed by 90 days of conditioning at room temperature (20 ± 2 °C) and 60 ± 5% humidity. Target temperatures were set to 20, 100, 200, 300, 500, and 700 °C, with a low heating rate of 1 °C/min. After heating, the specimens were evaluated for mass loss, compressive strength, UPV, and modulus of elasticity, and the experimental results were obtained by averaging the results obtained from three specimens.

### 2.2. Materials

**Figure 3:**
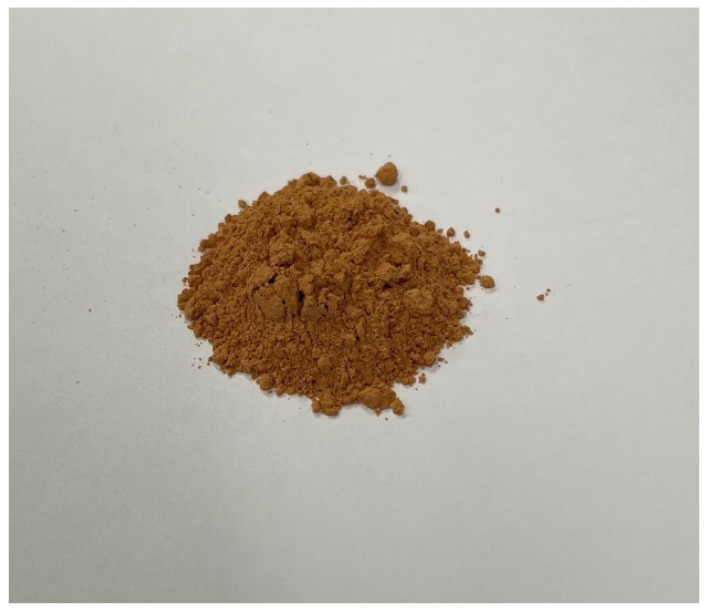
Non-sintered hwangto.

Table 3 outlines the physical and chemical properties of the materials used in this study. For cement, type I ordinary Portland cement with a density of 3150 kg/m^3^ and fineness of 320 m^2^/kg was used, and NSH with a density of 2500 kg/m^3^ and fineness of 330 m^2^/kg was mixed as a cement replacement material (Figure 3). The previous study used sintered hwangto for its reactivity with cement, but because of the CO_2_ emissions generated during the firing process, the use of hwangto was considered unsuitable for environmental benefits. Thus, in this study, NSH was used for mixing. Crushed granite aggregates with a density of 2680 kg/m^3^, a fineness modulus of 7.03, absorption of 0.68%, and a maximum size of 20 mm were used as coarse aggregates. River sand with a density of 2540 kg/m^3^, a fineness modulus of 2.54, and absorption of 1.6% was used as fine aggregate. To improve the workability deterioration resulting from the water absorption effect of NSH, a polycarboxylic-based acid was used as a superplasticizer. The NSH had lower CaO content than cement but higher SiO_2_ and Al_2_O_3_ concentrations.

### 2.3. Mix Proportions

Table 4 shows the mix proportions of the NC and NSHC used in this study; NC without NSH and NSHC with NSH are presented separately. The replacement ratio of NSH was 15 and 30% of cement, and W/B ratios of 0.41 and 0.33 were used in the experiment. In the “Mix ID” column, the numbers after NC and NSHC represent W/B, and “−15” and “−30” represent the ratios of NSH replacement for cement. The same types of coarse aggregate and fine aggregate were used for mixing at all levels.

### 2.4. Heating and Test Methods

**Figure 4:**
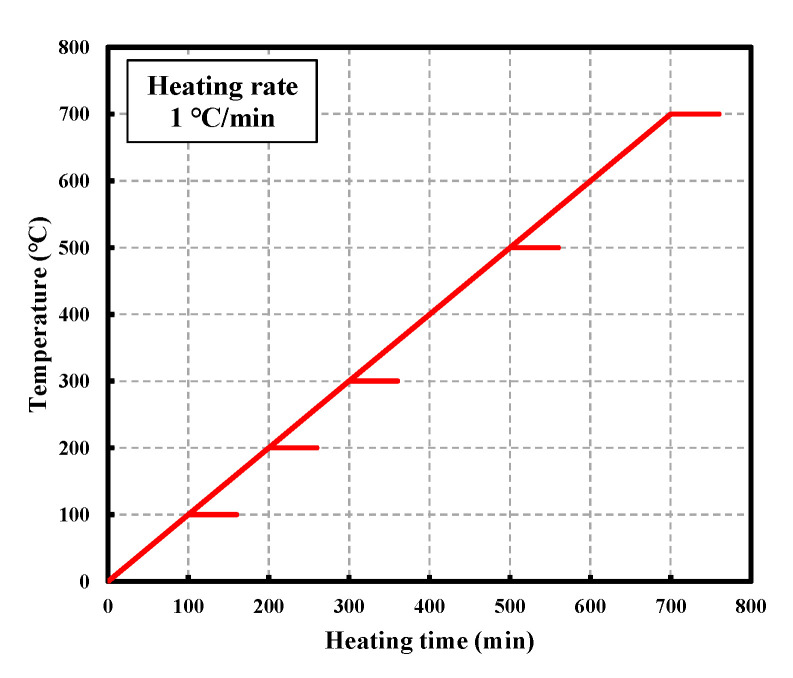
Heating curves.

In this study, the test specimen was heated using an electric heating furnace. Figure 4 presents the heating curves used in this study. The target temperatures were set to 20, 100, 300, 500, and 700 °C, with a low heating rate at 1 °C/min according to the recommendations in RILEM 129-MHT (test methods for mechanical properties of concrete at high temperatures). To ensure the application of uniform heating through the interior and exterior of the specimen, the temperature was maintained for 60 min after reaching the target level. The specimens were heated, then air cooled to measure their mechanical properties.

**Figure 5:**
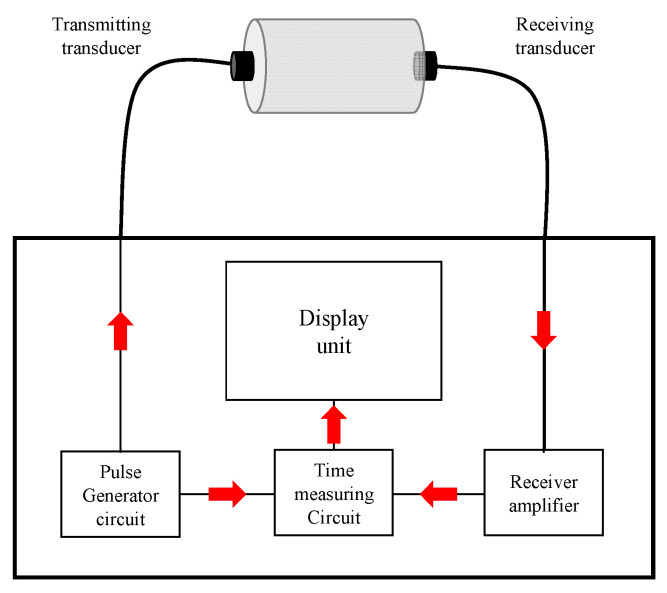
Ultrasonic pulse velocity test.

Table 5 shows the test methods for mechanical properties. The compressive strength of the specimen and modulus of elasticity were measured in accordance with ASTM C39/39M [30] and ASTM C469 [31], respectively. Figure 5 shows the UPV measurement mechanism, which is in accordance with ASTM C597 [32]. An electrical signal is transmitted from a pulse generator circuit, then from a “transmitting transducer” to a mechanical signal and to a “receiving transducer”. After changing the mechanical signal that reaches ‘receiving transducer’ from ‘receiving amplifier’ to an electrical signal, it is calculated based on the ‘time measuring circuit’ and displayed in the ‘display unit’. After measurement, UPV was calculated using Equation (1) as shown in Table 5, and all residual mechanical properties following high-temperature exposure were calculated using Equation (2).

## 3. Results and Discussion

### 3.1. Mass Loss

**Figure 6:**
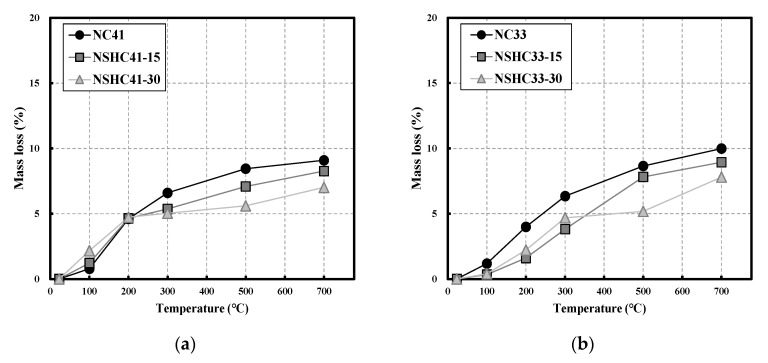
Mass loss of NC and NSHC after high-temperature exposure: (**a**) W/B 41; (**b**) W/B 33.

Figure 6 shows the mass loss of NC and NSHC following exposure to high temperatures by W/B. In the case of W/B 41 (Figure 6a), a mass loss rate of 4.67% was observed at all levels up to 200 °C, and at higher temperatures, a significant difference was observed depending on the NSH replacement ratio. NC 41 showed a mass loss of approximately 6.6% at 300 °C, whereas NSHC41-15 and NSHC41-30 showed a mass loss of 5.2%. For temperature ranges above 300 °C, NC41 showed a consistently high mass loss rate and different behavior depending on the NSH replacement ratio. At 500 °C, mass loss was approximately 26% higher in NSHC41-15 than in NSHC41-30, and at 700 °C, mass loss decreased as the NSH replacement ratio increased.

For W/B 33 (Figure 6b), NC33 showed a higher mass loss (%) than that of the specimens with NSH mixed in all temperature ranges. Similar to NC41, the mass loss was 6.34% up to 300 °C, and at 700 °C, the mass loss increased to approximately 10%. NSHC33-30 showed a by 23% higher mass loss than NSHC33-15 up to 300 ℃, but at higher temperatures, NSHC33-15 showed a higher mass loss value. NSHC33-15 showed a higher mass loss (%) than NSHC33-30 by approximately 51% at 500 °C and approximately 20% at 700 °C. Additionally, the dehydration of the chemically adsorbed water in cement occurs at temperatures below 600 ℃, and the decomposition of C-S-H calcium carbonate occurs above 600 °C. The mass loss (%) for NSHC with lower cement content than NC is relatively small, and it decreases as NSH replacement increases. Compared to the trend at 500 °C, the difference in mass loss (%) between NC and NSHC shows a decreasing trend at 700 °C. This is attributed to the effect of increased voids owing to the decomposition of silicon-aluminum hydrate of calcium carbonate (CaO_3_) and clay in NSH [33,34].

### 3.2. Residual Compressive Strength

**Figure 7:**
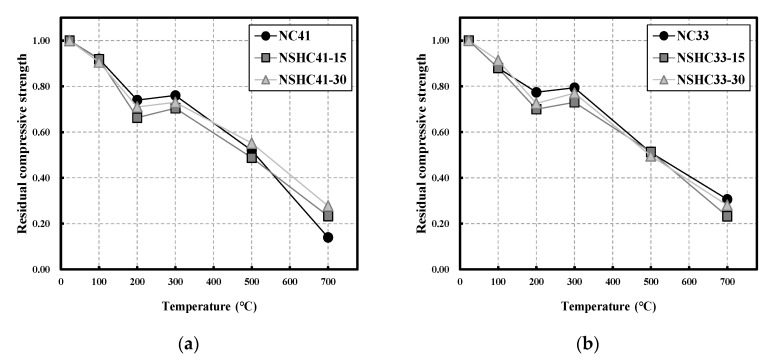
Residual compressive strength of NC and NSHC after high-temperature exposure: (**a**) W/B 41; (**b**) W/B 33.

Figure 7 shows the residual compressive strength with different W/B values after high-temperature exposure of NC and NSHC. All specimens showed a slight decrease in compressive strength for temperatures up to 200 °C, and the residual compressive strength of NSHC was approximately 3–8% lower than that of NC without NSH. Overall, there was a slight increase in compressive strength at 300 °C, which can be attributed to the complex effects of the expansion of coarse aggregates, vapor pressure, and rehydration reaction inside the concrete as reported in previous studies [35,36,37]. In the case of NC, the residual compressive strength was higher than that of NSHC, owing to the effect of the mixed cement content. However, compared to the specimen with 15% NSH replacement, the specimen with 30% NSH replacement showed high residual compressive strength despite lower cement content. Hwangto loses free water, absorbed water, and structural water at temperatures below 400 °C, resulting in internal shrinkage and structural deformation, which ware reflected as lower residual compressive strength than that of NC [38,39]. However, 30% NSH replacement results in higher residual compressive strength than that of 15% NSH replacement because of the effect of increasing cohesion by evaporation of water present on the rough surface of hwangto and the role of hwangto filling the voids left after water evaporation [40].

At temperatures above 300 °C, compressive strength showed a continuously decreasing trend. At 500 °C, all specimens with W/B 41 showed residual compressive strength of approximately 0.52, whereas those with W/B 33 showed residual compressive strength of approximately 0.51, exhibiting similar values overall. However, the residual compressive strength differed with W/B at 700 °C. NC41 showed residual compressive strength of approximately 0.14, whereas that of NSHC41-30 and NSHC41-15 was approximately 0.23 and 0.28, respectively, indicating notably higher residual compressive strength when mixed with NSH. Conversely, NC33 showed a residual compressive strength of approximately 0.31, whereas that of NSHC33-15 and NSHC 41-30 was approximately 0.23 and 0.28, respectively, which is comparable to that of NSHC with W/B 41. NSHC showed lower residual compressive strength than NC after exposure to low temperatures, but as the temperature increased, it showed similar or higher residual compressive strength than NC. According to Zhang et al., in hwangto, dehydration causes the density between particles to increase at temperatures between 400 and 1000 °C; scanning electron microscope (SEM) analysis revealed that in clay minerals, slight sintering caused the formation of aggregated structures [40]. According to Sung et al., some carbonate minerals found in hwangto decomposed above 600 °C and absorbed water from the air during the cooling process to form calcium hydroxide [38]. Therefore, it is inferred that NSH, as a cement replacement material, can be expected to have a positive effect on both safety following high-temperature exposure and environment sustainability.

### 3.3. Residual Ultrasonic Pulse Velocity

**Figure 8:**
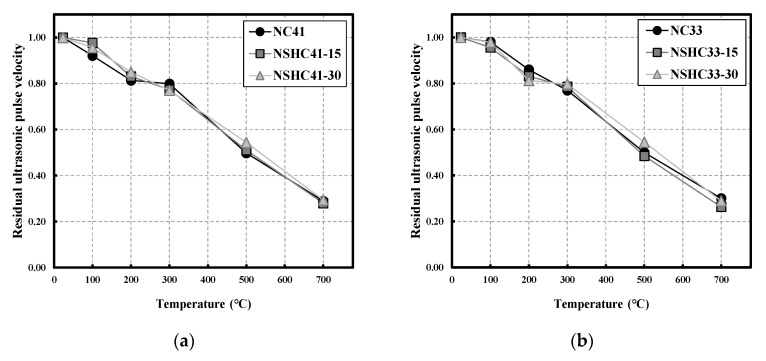
Residual ultrasonic pulse velocity on NC and NSHC after high-temperature exposure: (**a**) W/B 41; (**b**) W/B 33.

Figure 8 shows residual UPV by W/B following high-temperature exposure of NC and NSHC. The same W/B resulted in a similar trend for UPV regardless of NSH replacement, and as the temperature increased, UPV showed a continuously decreasing trend, unlike in the case of compressive strength [41]. At temperatures between 20 and 450 °C, active evaporation of free water and adsorbed water in concrete occurs, and voids are formed after water evaporation. Therefore, the ultrasonic wave changes from a liquid behavior to a gas behavior, in which lowers UPV. As discussed in Section 3.2, the reason for similar behavior between NC and NSHC in the abovementioned temperature range is that although the compressive strength decreased owing to shrinkage with evaporation of water, cohesion increased, improving the path of ultrasonic wave transmission.

At temperatures above 450 °C, the specimens completely dehydrated internally, the chemical components of cement decomposed, the interface between aggregate and cement paste weakened, and damage occurred to the matrix inside the cement. Accordingly, the sensitivity of UPV to microcracks and voids gradually decreases. At 500 and 700 °C, regardless of W/B, a similar residual UPV is exhibited, and residual UPV values of 0.52 at 500 °C and 0.28 at 700 °C were obtained. UPV is affected not only by the internal state of the specimen, such as cracks and voids, but also by the physical properties of the material such as density. Hwangto, which is less dense than cement, may contribute to a decrease in UPV, but at high temperatures, NSH forms aggregates, which improves UPV and results in similar behavior of residual UPV between NC and NSHC [40,41,42]. To accurately investigate the aforementioned phenomenon, further studies are required for a quantitative analysis of the correlation between the effects of NC cracks and voids and the effect of hwangto particles on UPV at high temperatures through microstructural analysis.

### 3.4. Residual Modulus of Elasticity

**Figure 9:**
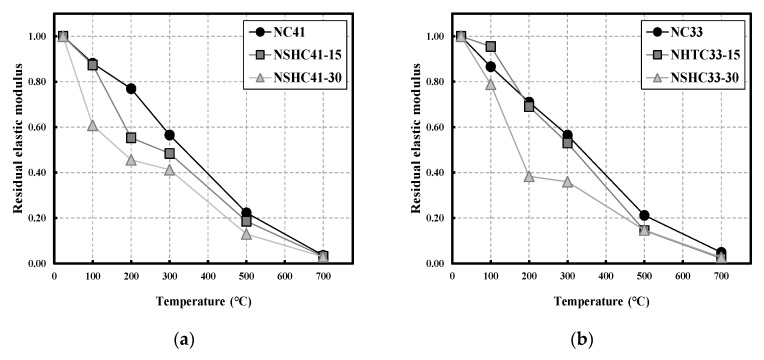
Residual elastic modulus of NC and NSHC after high-temperature exposure: (**a**) W/B 41; (**b**) W/B 33.

Figure 9 shows the modulus of elasticity with different values of W/B following high-temperature exposure of NC and NSHC. Similar to UPV, as the temperature increases, and the modulus of elasticity shows a linearly decreasing trend [43,44]. The residual modulus of elasticity was lower than other residual mechanical properties in all temperature ranges [45].

Additionally, the residual modulus of elasticity showed a decreasing trend as the NSH replacement increased. Figure 9a shows the residual modulus of elasticity for NC41 and NSHC41-15, which were both approximately 0.88 at 100 °C. At NSHC41-30, the value was 0.61, showing a very low residual modulus of elasticity. Figure 9b shows that the residual modulus of elasticity of NC33 at 100 °C was approximately 0.87, and the value was 0.95 for NSHC33-15, which is higher than that of NC33 without NSH. The residual modulus of elasticity for NSHC33-30 was 0.79, which is higher than that for NSHC41-30, but showed a very low value of approximately 0.38 at 200 °C. The residual modulus of elasticity decreases with increasing temperature and NSH replacement ratio. The residual modulus of elasticity of all specimens at 700 °C was approximately 0.04, indicating that elasticity was completely lost. The results show a similar trend to the experimental results of concrete exposed to high temperature [45,46,47], and with NSH replacement of 30%, a very low residual modulus of elasticity was shown at low temperatures compared to other materials. Therefore, it can be reasoned that when NSHC is used as a construction material, reinforcing bars should be considered.

### 3.5. Relative Mechanical Properties

**Figure 10:**
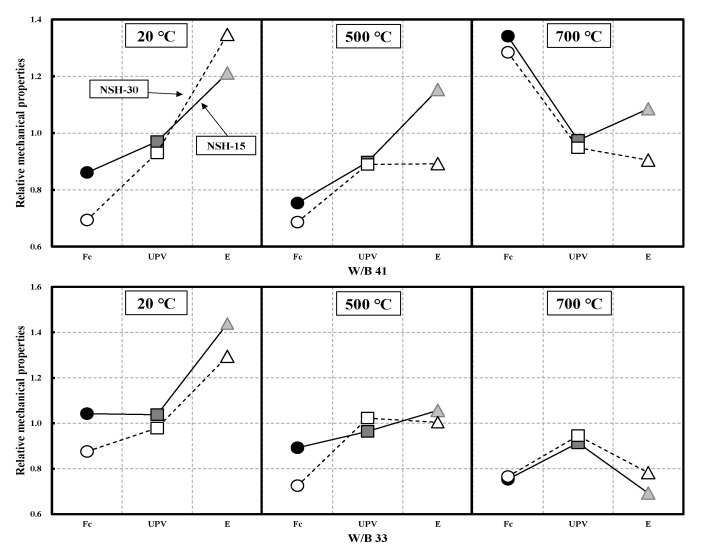
Relative mechanical properties according to replacement of NSH compared to NC by W/B.

Figure 10 shows the relative mechanical properties according to the replacement of NSH compared to NC by W/B. For W/B 41, NSHC demonstrated lower strength compared to NC with increasing NSH replacement before heating (20 °C) and at 500 °C, but at 700 °C, the relative strength was higher by 34% for 15% NSH replacement and by 28% for 30% NSH replacement. At all temperatures, regardless of NSH replacement, the UPV of NSH was approximately 93% compared to NC, demonstrating a similar trend. Regarding the modulus of elasticity, before heating, the NSHC had a higher value than NC with increasing NSH replacement. However, for 15% NSH replacement at 500 and 700 °C, the relative modulus of elasticity was higher by 12% compared to NC, but for 30% NSH replacement, it was lower by 10% compared to NC.

For W/B 33, before heating (20 °C), in the case of 15% NSH replacement, compressive strength and UPV of NSHC were approximately 4% higher than those of NC, and the modulus of elasticity was 20% higher than that of NC. In the case of 30% NSH replacement, the compressive strength was low, but UPV showed a similar value, and the modulus of elasticity was significantly higher by 35% compared to NC. However, at 500 and 700 °C, the compressive strength of NSHC was lower than that of NC, and UPV was similar between NT and NSHC. Regarding the modulus of elasticity, before heating, the NSH replacement had a higher value than NC. However, in the case of high-temperature exposure, the modulus of elasticity showed a rapid decrease.

### 3.6. Correlation between Residual Compressive Strength and Ultrasonic Pulse Velocity

**Figure 11:**
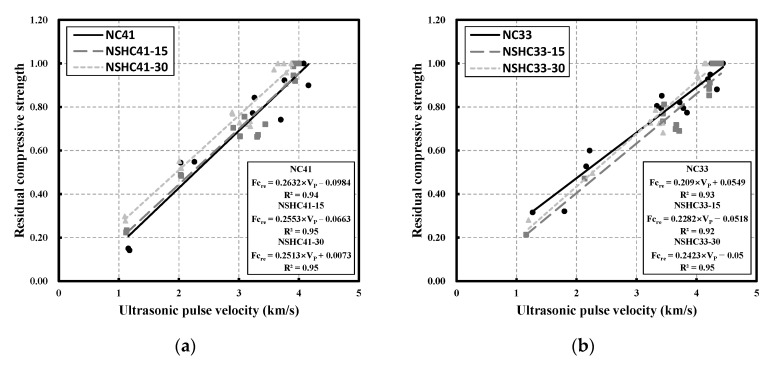
Correlation between residual compressive strength and ultrasonic pulse velocity in NC and NSHC after high-temperature exposure: (**a**) W/B 41; (**b**) W/B 33.

Figure 11 shows the correlation between residual compressive strength and UPV in NC and NSHC after high-temperature exposure. Previous research has shown that compressive strength and UPV are correlated in several ways, such as by exponential functions, linear equations, and quadratic equations [48,49,50,51]. According to the correlation analysis results, the correlation based on a linear equation is appropriate, and the trend of UPV showing a linear decrease with increased temperature may have had an effect on the form of correlation. Figure 11a shows the correlation between NC and NSHC with W/B 41. NC41 and NSHC41-15 showed similar results, with correlation coefficients (R^2^) of 0.94 and 0.95, respectively. Conversely, the linear regression graph of NSHC41-30 showed a result that was greater than the other two graphs by approximately 16%, which is thought to be a result of the effect of high residual compressive strength with increasing temperature (Figure 7).

Figure 11b illustrates the result of the correlation analysis for NC and NSHC with W/B 33. The high UPV range showed a similar trend, whereas the relatively low UPV range showed a distinct difference. This indicates that as the temperature increases, NSH replacement has a significant effect on the result. With a smaller UPV range, NC33 values are greater than NSHC33-15 and NSHC33-30 by approximately 21% and 12%, respectively, and NSHC33-15 showed the lowest values among the three graphs. However, NSHC33-15 and NSHC33-30 showed similar trends in the very low UPV range.

**Figure 12:**
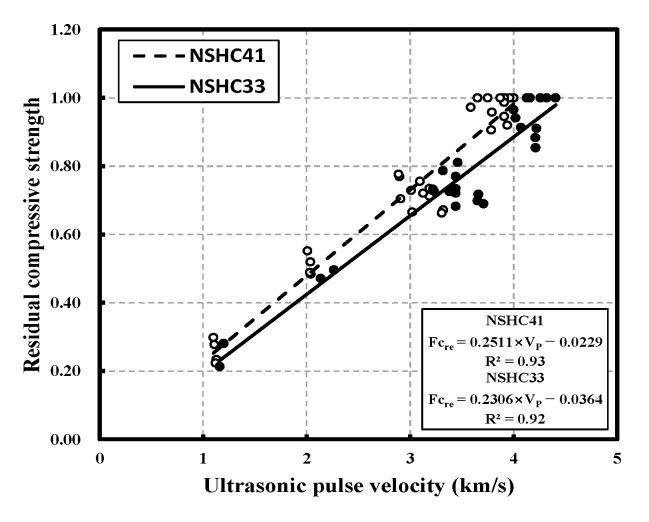
Correlation between residual compressive strength and ultrasonic pulse velocity in NSHC after high-temperature exposure.

Figure 12 shows the correlation between residual compressive strength and UPV in NSHC following high-temperature exposure and presents a prediction model for evaluation of the residual compressive strength of NSHC following high-temperature exposure using UPV analysis. Similar to the model described above, a prediction model in the form of a linear equation was proposed. There was no significant difference in the trend of UPV with increasing temperature (Figure 8), but the residual compressive strength showed a significant difference (Figure 7). The equation proposed in this study sets UPV as the independent variable and residual compressive strength as the dependent variable. However, even if the independent variable does not change, there is a difference in the dependent variable, and it is determined that a prediction model different from NC should be used for NSHC mixed with NSH. The prediction equation proposed in this study was classified by W/B, and data were analyzed without considering NSH replacement. R^2^ for NSHC41 and NSHC33 was high, with values 0.93 and 0.92, respectively, and NSHC41 values were greater than those of NSHC33 by approximately 11%.

## 4. Conclusions

In this study, we investigated the high-temperature properties of NC and NSHC, and through UPV analysis following high-temperature exposure, a prediction model for compressive strength of NSHC was developed.

(1) Mass loss decreased as temperature increased, whereas NSH replacement increased. Significant differences were observed at temperatures above 500 °C, with NSHC41-15 demonstrating a higher mass loss of approximately 26% compared to NSHC41-30, and NSHC33-15 demonstrated a higher mass loss of approximately 51% compared to NSHC33-30.
(2) The residual compressive strength of NSHC41-15 and NSHC41-30 at 700 °C was 0.23 and 0.28, respectively, which is higher than that of NC41; all specimens with W/B 33 showed similar residual compressive strength. This is believed to be the effect of the formation of aggregated structures according to the increase in cohesion following the evaporation of water on the surface of the NSH and the slight sintering of clay minerals.
(3) Regardless of NSH replacement, residual UPV showed a linearly decreasing trend with increasing temperature. This is because hwangto, which has a low density, may lower UPV, whereas NSH, which forms aggregates at high temperatures, has a positive effect on UPV.
(4) The residual modulus of elasticity for NSHC41-30 at 100 °C was 0.61 and 0.38 for NSHC33-30 at 200 °C. Therefore, the modulus of elasticity showed a significant decrease with increasing NSH replacement, and with rising temperature, it also decreased with increasing NSH replacement.
(5) The analysis of the correlation between residual compressive strength and UPV following high-temperature exposure revealed that for W/B 41, NC41 and NSHC41-15 showed a similar trend, but NSHC41-30 had an approximately 16% higher value than the other two specimens. In the case of W/B 33, as the temperature increased, NC33 showed values higher than those of NSHC33-15 and NSHC33-30 by approximately 21% and 12%, respectively. Finally, following high-temperature exposure of NSHC, an equation for predicting compressive strength was proposed by W/B using UPV analysis. For NSHC41 and NSHC33, the correlation between residual compressive strength and UPV was strong, with the following correlation coefficients (R^2^): 0.93 and 0.92, respectively.

## Figures and Tables

**Table 1:** Previously proposed equations for estimating compressive strength by ultrasonic pulse velocity [26,27,28,29].

Researcher	Admixture	Equation
G. Sua-iam	Limestone powder	$F_{c}=18.311\times \mathrm{Vp}-29.114$
S. K. Rao	Fly ash	$F_{c}=1.526\times e^{0.761\times \mathrm{Vp}}$
M. Shariq	GGBF 0%	$F_{c}=0.294\times e^{1.044\times \mathrm{Vp}}$
	GGBF 20%	$F_{c}=0.199\times e^{1.14\times \mathrm{Vp}}$
	GGBF 40%	$F_{c}=0.1\times e^{1.29\times \mathrm{Vp}}$
	GGBF 60%	$F_{c}=0.175\times e^{1.16\times \mathrm{Vp}}$

**Table 2:** Experimental parameters.

ID	Replacement Rate of NSH ^(1)^	W/B	Curing	Heat Method	Test Item
NC	0% 15% 30%	0.41 0.33	Water curing Room temperature (20 ± 2 °C) Humidity (60 ± 5%)	20, 100, 200, 300, 500, 700 °C (1 °C/min)	Compressive strength Ultrasonic pulse velocity Modulus of elasticity
NSHC					

^(1)^ NSH: non-sintered hwangto.

**Table 3:** Physical and chemical properties of the materials.

Property	Cement (Type I Ordinary Portland Cement)	Non-Sintered Hwangto	Coarse Aggregate (Crushed Granite)	Fine Aggregate (River Sand)	Super Plasticizer
Density (kg/m^3^)	3150	2500	2680	2540	Polycarboxylic-based acid
Fineness (m^2^/kg)	320	330	-	-	
Fineness modulus	-	-	7.03	2.54	
Absorption (%)	-	-	0.68	1.6	
Maximum size (mm)	-	-	20	-	
Chemical Composition (%)					
CaO	60.34	0.39			
SiO_2_	19.82	40.0			
Al_2_O_3_	4.85	32.9			
Fe_2_O_3_	3.30	7.79			
MgO	3.83	1.54			
SO_3_	2.88	-			
K_2_O	1.08	0.76			
Others	0.86	16.62			
L.O.I	3.02	13.7			

**Table 4:** Mix proportions of the NC and NSHC.

Mix ID	NC41	NSHC41-15	NSHC41-30	NC33	NSHC33-15	NSHC33-30
Water/Binder	0.41	0.41	0.41	0.33	0.33	0.33
Sand/aggregate (%)	46.0	46.0	46.0	43.0	43.0	43.0
Water (kg/m^3^)	165	165	165	165	165	165
Cement (kg/m^3^)	400	340	280	500	425	350
Non-sintered hwangto (kg/m^3^)	-	60	120		75	150
Fine aggregate (kg/m^3^)	799	794	788	711	705	699
Coarse aggregate (kg/m^3^)	956	950	943	961	953	944

**Table 5:** Testing of residual mechanical properties.

Test Item	Test Method	Equation (1)	Equation (2)
Compressive strength	ASTM C39/C39M	$V_{p}=\frac{L}{t}$	$M_{\mathrm{re}}=\frac{M_{R}}{M_{T}}$
Modulus of elasticity	ASTM C469		
Ultrasonic pulse velocity	ASTM C597	$V_{p}$: ultrasonic pulse velocity(m/s) L: distance(m) t: time(s)	$M_{\mathrm{re}}$: residual mechanical properties $M_{R}$: mechanical properties at room temperature $M_{T}$: mechanical properties at high temperature

## Data Availability

The data presented in this study are available upon request from the corresponding author.
